# The validity and reliability of the PHQ-9 on screening of depression in neurology: a cross sectional study

**DOI:** 10.1186/s12888-021-03661-w

**Published:** 2022-02-09

**Authors:** Yajing Sun, Zhifei Kong, Yuqing Song, Jing Liu, Xilin Wang

**Affiliations:** Peking University Institute of Mental Health, Peking University Sixth Hospital, Key Laboratory of Mental Health, National Health Commission (Peking University), National Clinical Research Center for Mental Disorders (Peking University Sixth Hospital), No. 51 Huayuan North Road, Haidian District, Beijing, 100191 China

**Keywords:** Depression, Patient Health Questionnaire-9 (PHQ-9), Neurological disorders, Validity, Reliability

## Abstract

**Background:**

This study aimed to explore the validity and reliability of the Patient Health Questionnaire-9 (PHQ-9) on screening of depression among patients with neurological disorders, and to explore factors influencing such patients.

**Methods:**

In this study, 277 subjects who were admitted to the department of neurology of our hospital due to different neurological disorders completed the PHQ-9 questionnaire. The Mini-International Neuropsychiatric Interview (MINI) and Hamilton Rating Scale for Depression (HAMD) were employed to evaluate the depressive symptoms of patients who completed the PHQ-9 questionnaire. The internal consistency, criterion validity, structural validity, and optimal cut-off values of PHQ-9 were evaluated, and the consistency assessment was conducted between the depression severity as assessed by PHQ-9, HAMD and MINI. Logistic regression analysis was used to calculate the risk factors of depression.

**Results:**

The Cronbach’s *α* coefficient of the PHQ-9 was 0.839. The Pearson’s correlation coefficient among the 9 items of the PHQ-9 scale was 0.160 ~ 0.578 (*P* < 0.01), and the Pearson’s correlation coefficient between each item and the total score was at the range of 0.608 ~ 0.773. Taking the results of MINI as the gold standard, the area under the receiver operating characteristic (ROC) curve of the PHQ-9 results for all the subjects (*n* = 277) was 0.898 (95% confidence interval (CI): 0.859 ~ 0.937, *P* < 0.01). When the cut-off score was equal to 5, the values of sensitivity, specificity, and the Youden’s index were 91.2, 76.6%, and 0.678, respectively. Multivariate logistic regression analysis showed that the influence of unemployment on the occurrence of depression was statistically significant (*P* = 0.027, OR = 3.080, 95%CI: 1.133 ~ 8.374).

**Conclusions:**

The application of PHQ-9 for screening of depression among Chinese patients with neurological disorders showed a good reliability and validity.

## Introduction

Mental disorder, also called mental illness or psychiatric disorder, is a behavioral or mental pattern that causes significant distress or impairment of personal functioning [1]. Depression, as an important mental disorder, was ranked as the third cause of burden of disease worldwide in 2008 and may rank first by 2030 [2]. Depression, which often accompanies multiple diseases, imposes serious health and economic burdens to society [3, 4]. It is highly prevalent among patients suffering from various chronic conditions [5]. There are multiple ways in which depression can be identified. As for mild forms of depression, it may recover without much clinical assistance or only need primary care. However, major depression, especially severe depression, requires advanced care and early identification [6]. Identifying cases with depression that require advanced care is not only a main challenge to primary care, but also for clinicians, especially for non-psychiatric physicians.

Neurology and psychiatry are often closely related. There are several factors influencing the incidence of depression on patients with neurological disorders, and controversial results were reported. A previous study showed that epilepsy was an independent risk factor for depression [7]. Scholars found that severe motor function, dyskinesia, poor sleep quality, and cognitive impairment were independent predictors of depression in Parkinson’s disease (PD) patients who were admitted to department of neurology [8, 9]. However, depression in patients with neurological disorders and associated risk factors need further clinicians’ and scholars’ attention. Training non-psychiatric doctors to successfully identify patients with severe depression through the method of mental examination may resolve the mentioned challenge, while it is costly and time-consuming [10, 11]. A large number of health care systems have employed screening tools, such as the Self-rating Depression Scale (SDS) [12], the Structured Clinical Interview for DSM-IV Axis I Disorders (SCID-I) [13], Composite International Diagnostic Interview (CIDI) [14], the Mini-International Neuropsychological Interview (MINI) [15], the Cornell Scale for Depression in Dementia (CSDD) [16], and the Hamilton Rating Scale for Depression (HAMD) [17, 18] to evaluate severity of depressive symptoms. However, such tools are not optimal as they (1) tie up significant resources, such as trained professionals [14, 15, 17], (2) cannot be used for diagnosis but with many items needed to be evaluated [12], or (3) can only be used for diagnosis of specific patients [16].

Patient Health Questionnaire-9 (PHQ-9) was derived from the depression part in the Patient Health Questionnaire (PHQ) compiled by Spitzer et al. in 1999 [19]. PHQ-9 was recommended by the Diagnostic and Statistical Manual of Mental Disorders, Fifth Edition (DSM-5). Response options on the items range from ‘not at all’ (0-point) to ‘nearly every day’ (3-point). The scale can not only screen for depression, but also show the severity of depression [20]. Because of its convenient use and good reliability and validity, it has been widely used for depression screening in the internal medicine department of primary hospitals. The depression screening of the elderly, patients with epilepsy, and stroke patients also had good reliability and validity [21–23].

However, there still lies some uncertainties to be explored. Different studies have shown that the optimal cut-off value of PHQ-9 varies in different populations. The PHQ-9 maker used a cut-off value of 10, with the sensitivity 88% and the specificity 88% [20]. In 2012, a meta-analysis showed that the optimal cut-off value of PHQ-9 was 8-11 [24]. The best cut-off value of PHQ-9 for diagnosing depression still needs further discussion. The original researchers of PHQ-9 used 5, 10, 15, 20 as the demarcation values for mild depression, moderate depression, severe depression, and very severe depression [25]. If PHQ-9 is used in different populations, the screening cut-off value changes, then the corresponding evaluation of depression severity may also change, which has certain guiding significance for treatment. What’s more, the PHQ-9 may lack some symptoms that are meaningful to the depressive patient, and the description of the symptoms is not clear enough [26]. For example, patients with depression will regard abnormal perception, depersonalization, isolation, loneliness, and physical sensations (such as tremor, fatigue, restlessness, nausea, inability to relax, etc.) as meaningful or strong feelings of their depression. However, these symptoms are not reflected in the PHQ-9. As a self-rated scale, the PHQ-9 still needs to be completed by doctor involved in assisting patients to reduce confusion and to express their inner feelings accurately. Regarding the use of the PHQ-9, there lies several problems, such as the cut-off value, the inconsistency of reliability and validity when use in neurology, and the language expression which may need to be adjusted. PHQ-9 needs to be further explored in patients with neurological disorders in order to improve its diagnostic value.

In the present study, general data of patients who were admitted to the Department of Neurology of an affiliated hospital of Peking University due to different neurological disorders were collected, and the PHQ-9 questionnaire was distributed among those patients. One trained psychiatrist used the Mini-International Neuropsychiatric Interview (MINI) to evaluate the depressive symptoms of patients who completed the PHQ-9 questionnaire. Two senior psychiatrist used the HAMD to assess the severity of depression. The internal consistency, criterion validity, structural validity, and optimal cut-off values of PHQ-9 were evaluated, and the consistency assessment was conducted between the depression severity as assessed by PHQ-9 and HAMD. We also explored factors (e.g., age, gender, medical insurance, course of disease, work conditions, etc.) influencing such patients and discussed their influences comprehensively.

## Methods

### The aim, design and setting of the study

We aimed to explore the validity and reliability of the Patient Health Questionnaire-9 (PHQ-9) in the neurology ward when screening depression. This is a cross-sectional study. We hoped to screen all inpatients in neurology for depression and its severity using the PHQ-9. This study was approved by the Ethics Committee of Peking University Six Hospital (No.2009025).

### Study subjects

**Fig. 1:**
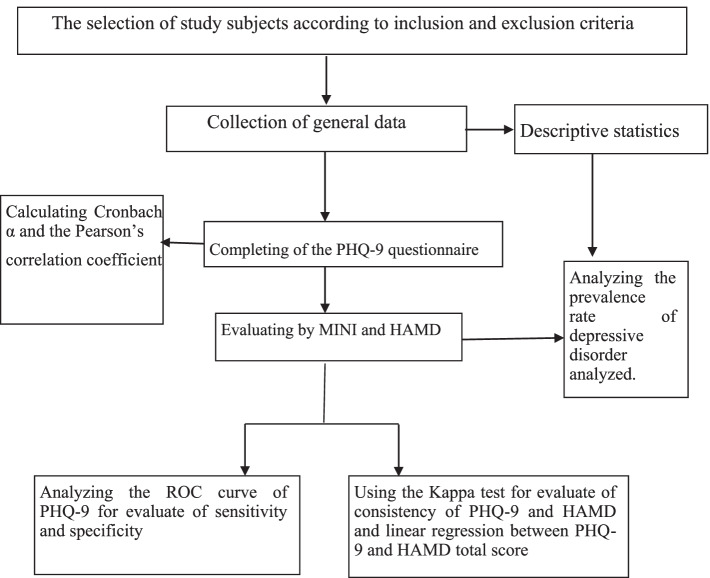
Flow chart

From January 2016 to June 2016, patients with depression who suffered from neurological disorders were admitted to the Neurology Department of an affiliated hospital of Peking University (Beijing, China). Inclusion criteria were as follows: i) patients (age ≥ 18 years old) from the Neurology Department of an affiliated hospital of Peking University, ii) absence of a significant cognitive impairment (Mini-Mental Status Examination > 21) [27, 28], and iii) patients who signed the written informed consent form prior to commencing the study. Exclusion criteria were as follows: i) patients with speech dysfunction and hearing impairment, who could not complete the questionnaire, or ii) patients who aged < 18 years old. A total of 300 questionnaires were distributed among eligible patients, and a total of 290 questionnaires were returned, accounting for 96.7%. Those patients received MINI and Hamilton Rating Scale for Depression (HAMD), and the total number of patients who completed all the survey was 277. A self-edited questionnaire was designed to collect patients’ general data, including patients’ name, gender, age, ethnicity, marriage status, work experience, treatment costs, course of disease, diagnostic method, etc. The flowchart of patients’ selection is shown in Fig. 1.

### Research tools

#### PHQ-9

The PHQ-9 is a 9-question instrument given to patients in a primary care setting to screen the presence and severity of depression. This is a self-rating scale. The results of the PHQ-9 are used to make a depression diagnosis according to the DSM-IV (Diagnostic and Statistical Manual of Mental Disorders–Fourth Edition) criteria. Here, the PHQ-9 was formulated based on DSM-IV to understand how often patients have been bothered by symptoms of depression in the period of two weeks (0 point = never, 1 point = a few days, 2 point = more than half of the days, 3 point = almost every day). Each item was scored on a scale of 0-3, with a total score ranging from 0 to 27. Based on these scores, depressive symptoms could be divided into “none or minimum” (0-4), “mild” (5-9), “moderate” (10-14), “moderately severe” (15-19), and “severe” (20-27).

#### MINI (Chinese version)

As a semi-fixed diagnostic tool developed by a number of Chinese scholars, the MINI is a short structured interview used to diagnose 16 axis I DSM-IV and ICD-10 (International Classifications of Diseases and Related Health Problems, Tenth Revision) disorders [29]. A previous research showed that the Chinese version of MINI had good reliability and validity, as well as high sensitivity and specificity for depressive disorders. The current study used the evaluation results of depression in the MINI as the “gold standard” to assess the validity of PHQ-9. We defined “1” = have depression and “0” = have no depression. This scale was completed through interviews. The depression diagnosis was made by 1 psychiatrist, deputy chief physician.

#### HAMD

The HAMD [18] is a 17-item instrument that was designed to measure frequency and intensity of depressive symptoms in individuals with major depressive disorders. HAMD possesses a good reliability and validity. It comprises of 17 items, and was previously grouped into 5 structural factors (i.e., anxiety/somatization, mental disorders, retardation symptoms, sleep disturbances, and weight loss) by Cleary and Guy [30]. The higher the score, the more severe the symptoms. The following ranges for the HAMD were recommended: no depression (0–7); mild depression (8–16); moderate depression (17–23); and severe depression (≥24). In our research, two physicians assessed the severity of the patients’ depression using HAMD.

### Statistical analysis

Sample size:

$$\mathrm{n}={\left[\frac{57.3{Z}_{\alpha /2}}{\sin^{-1}\left(\frac{\delta }{\sqrt{p\left(1-p\right)}}\right)}\right]}^2$$

Explanation: Z_α/2_ is the Z value of cumulative probability in normal distribution (Z_0.05 / 2_ = 1.960); δ is the allowable error; α is the inspection level; P is sensitivity or specificity.

Taking the 10 points recommended by the original maker of PHQ-9 as the screening cut-off value, a study covering 6000 subjects reported that the sensitivity was 88% and the specificity was 88% [20]. Therefore, it was expected that the sensitivity and specificity of this test would be 88% both. We took 0.05 as the significance level α, 0.08 as the allowable error. According to the formula, the number of samples in the case group was 63 and that in the control group was 63, too. The incidence of depression in hospitalized patients in neurology department was 25.0-50% [9, 31, 32]. This experiment predicted that the prevalence rate was 25%. It was estimated that the number of PHQ-9 questionnaires issued at least should be 63 / 0.25 = 252. Taking into account the 10% loss to follow-up rate, we set the sample size as 300 cases.

SPSS 22.0 statistical software (IBM, Armonk, NY, USA) was used to perform statistical analysis, and descriptive statistics were used for expressing general data and other related descriptions. The receiver operating characteristic (ROC) curve was employed to analyze the validity, sensitivity, specificity, positive predictive value, negative predictive value, and Youden’s index of the PHQ-9, so as to find the best diagnostic cut-off score. Based on the cut-off points, consistency analysis between the severity of depression obtained by PHQ-9 and HAMD revealed a Kappa score. The linear regression analysis of PHQ-9 and HAMD was performed to obtain the PHQ-9 cut-off score for depressive symptoms with different diversities. The intraclass correlation coefficient (ICC) and Cronbach’s alpha coefficient were used to assess internal consistency. The confirmatory factor analysis was employed to analyze the structural validity of the scale. We used logistic regression analysis to explore risk factors of depression.

## Results

### The participants’ general data

A total of 277 participants were involved (Table 1), including 181 (65.3%) male and 96 (34.7%) female cases. The participants aged 18-88 years old, with a mean age of 60.56 ± 15.53 years old. Regarding ethnicity, 264 (95.3%) were Han people and 13 (4.7%) cases were from other ethnicities. Table 1 presented the participants’ demographic characteristics. In terms of participants’ marital status, 252 (91.0%), 2 (0.7%), 4 (1.4%), and 19 (6.9%) cases were married, divorced, widowed, and unmarried, respectively. Regarding occupation, 83 (30%), 112 (40.4%), and 29 (10.5%) patients were in-service, retired, and unemployed, respectively, and 53 (19.1%) patients had other professions. As for medical expenses, 271 (97.8%) cases were covered by health insurance, while 6 (2.2%) cases were at their own expenses. There were 209 (75.4%) cases of cerebrovascular diseases, 14 (5.1%) cases of peripheral neuropathy, and 54 (19.5%) cases with other diseases (e.g., cervical spondylosis, epilepsy, etc.). Besides, 205 (74.0%) and 30 (10.8%) patients’ course of disease was within 1 month, over 12 months, and that of 42 (15.2%) patients varied between 1 and 12 months.

**Table 1:** Sociodemographic data

	Number	Proportion
**Age**		
< 65 years old	152	54.9%
≥ 65 years old	125	45.1%
**Gender**		
Male	181	65.3%
Female	96	34.7%
**Ethnicity**		
Han	264	95.3%
Other ethnicities	13	4.7%
**Marital status**		
Married	252	91%
Divorced	2	0.7%
Unmarried	19	6.9%
Widowed	4	1.4%
**Occupation**		
Public servants	83	30%
Retired	112	40.4%
Unemployed	29	10.5%
Other professions	53	19.1%
**Expense categories**		
Reimbursement	271	97.8%
Self-supporting	6	2.2%
**Disease**		
Cerebrovascular diseases	209	75.4%
Peripheral neuropathy	14	5.1%
Other diseases	54	19.5%

### PHQ-9 scores, HAMD-17 scores and MINI results

The mean score of PHQ-9 in 277 cases was 5.27 ± 1.86. It was revealed that 166 (59.9%) patients had no depression, and 111 (40.1%) patients had depressive symptoms. Among them, 61 (22.0%), 22 (8.0%), 16 (5.8%), and 12 (4.3%) cases had mild, moderate, severe, and extremely severe-depression, respectively. The mean HAMD-17 score of 277 cases was 7.75 ± 2.83. According to HAMD-17 scores, 158 (57.0%) patients had no depression. Among 119 (43.0%) patients with depression, 82 (29.6%), 27 (9.8%), and 10 (3.6%) patients had mild, moderate, and severe-depression, respectively. MINI is fully structured to allow administration in about 15 to 20 min even by nonspecialized interviewers. It demonstrates good sensitivity, specificity, validity and reliability in the assessment of psychiatric disorders. The major depressive episode module was used in this study as a gold standard. Among 277 subjects who completed the MINI, 68 (24.5%) cases were diagnosed with depression, while 209 (75.5%) cases had no depression.

### Reliability

In order to investigate the reproducibility and consistency of PHQ-9, reliability coefficients as measured by Cronbach’s alpha were calculated. The Cronbach’s α coefficient for PHQ-9 was 0.839. When one of the items of PHQ-9 was deleted, the α coefficient was still between 0.806 ~ 0.839. The Pearson’s correlation coefficient among the 9 items of the PHQ-9 scale was at the range of 0.160 ~ 0.578 (*P* < 0.01), and the Pearson’s correlation coefficient between each item and the total score was within 0.608 ~ 0.773. The above-mentioned coefficients were statistically significant (*P* < 0.01) (Table 2).

**Table 2:** Correlation between items and between each item and the total score

	Item1	Item2	Item3	Item4	Item5	Item6	Item7	Item8	Item9	Total
Item1	1									
Item2	0.569^**^	1								
Item3	0.333^**^	0.343^**^	1							
Item4	0.558^**^	0.504^**^	0.373^**^	1						
Item5	0.351^**^	0.354^**^	0.358^**^	0.408^**^	1					
Item6	0.440^**^	0.529^**^	0.265^**^	0.330^**^	0.328^**^	1				
Item7	0.328^**^	0.339^**^	0.161^**^	0.350^**^	0.240^**^	0.205^**^	1			
Item8	0.510^**^	0.463^**^	0.309^**^	0.440^**^	0.330^**^	0.375^**^	0.497^**^	1		
Item9	0.399^**^	0.578^**^	0.272^**^	0.344^**^	0.279^**^	0.567^**^	0.160^**^	0.373^**^	1	
Total	0.762^**^	0.773^**^	0.608^**^	0.743^**^	0.610^**^	0.647^**^	0.517^**^	0.707^**^	0.621^**^	1

Note: **P < 0.01(Two-sided test)

### Validity

#### Construct validity

In this research, the eigenvalues of factor-1, factor-2, and factor-3 were 3.385, 1.248, and 1.050, with the corresponding the explanatory variances of 37.615, 13.868, and 11.661%, respectively. The cumulative interpretation variance of the three factors was 63.114%. For rotated component matrix of factor analysis, the coefficients of interest decline, fatigue, mental motor delay, difficulty in paying attention, emotional depression, and factor-1 were 0.736, 0.717, 0.701, 0.694, and 0.563, respectively. The coefficients of suicide and self-injury, inferiority and factor-2 were 0.806 and 0.758, respectively. The matrix coefficients of sleep disorder, eating disorder and factor-3 were 0.828 and 0.781, respectively (Table 3).

**Table 3:** Coefficients of scale entries and factors

Inventory items	Factor1	Factor2	Factor3
Lack of enthusiasm or interest in doing something	0.736	–	–
Feeling tired or unenergized	0.717	–	–
Slow or fidgety	0.701	–	–
You have trouble focusing on things	0.694	–	–
The mood is low, depressed, or disappointed	0.563	–	–
suicide and self-injury	–	0.806	–
Fee bad. It’s a shame to my family.	–	0.758	–
Difficulty in falling asleep, restless sleep, or excessive sleep	–	–	0.828
Loss of appetite or eating too much	–	–	0.781

#### Criterion validity

**Fig. 2:**
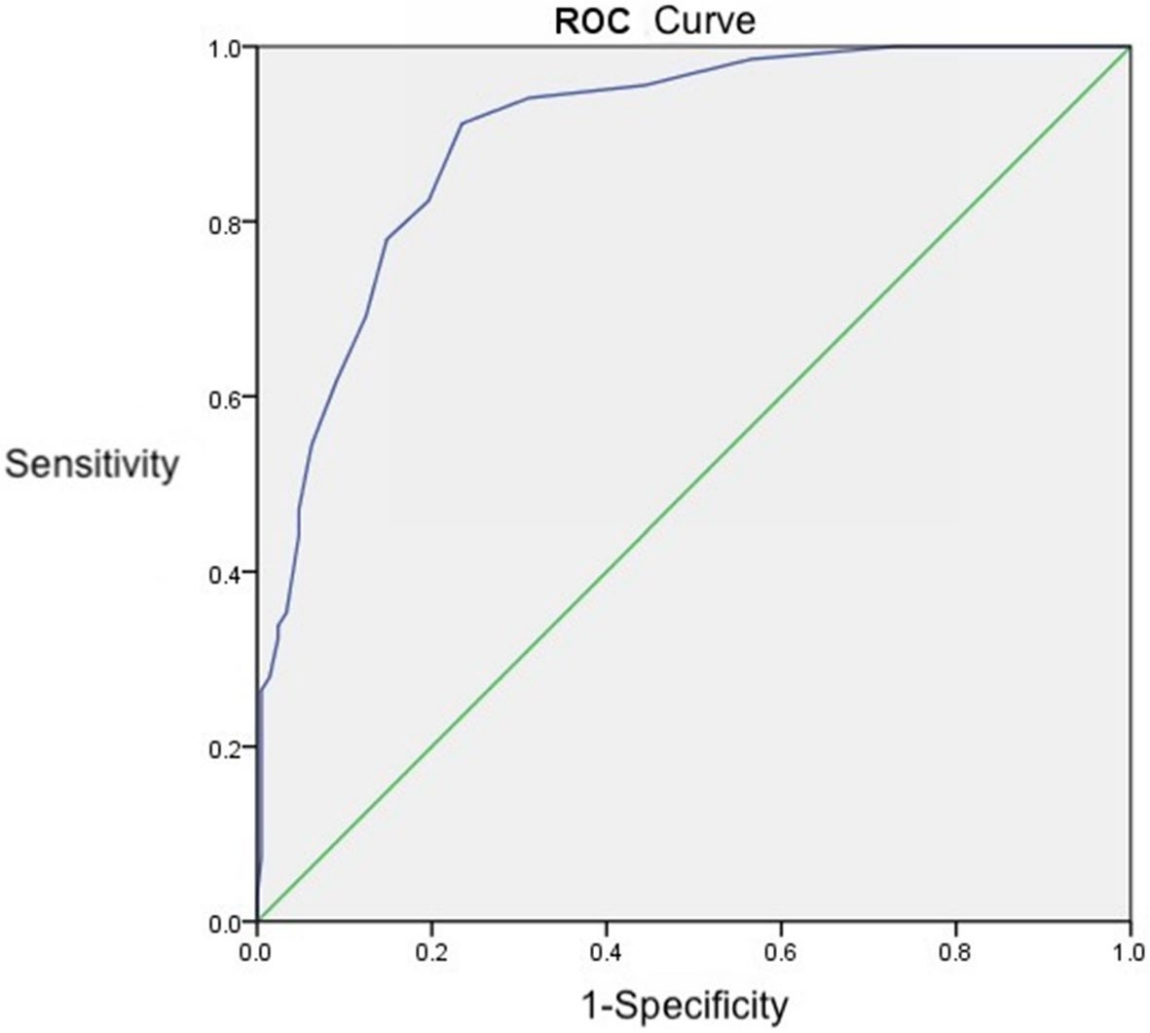
ROC Curve

Criterion validity was assessed by ROC curve. The PHQ-9 score simultaneously showing the highest sensitivity and specificity was evaluated using the ROC curve. PHQ-9’s accuracy was estimated by the area under the ROC curve (AUC). As shown in Fig. 2, the results of ROC curve analysis indicated that the AUC for PHQ-9 was 0.898 (95% confidence interval (CI): 0.859 ~ 0.937), which indicated that PHQ-9 possessed a good ability to identify depressive symptoms. When the cut-off scores were 3, 4, 5, 6, 7, 8, 9, and 10, the rates of sensitivity were 95.6, 94.1, 91.2, 82.4, 77.9, 69.1, 61.8, and 54.4%, the rates of specificity were 55.5, 68.4, 76.6, 80.4, 85.2, 87.1, 90.9, and 93.8%, and the values of Youden’s index were 0.511, 0.625, 0.678, 0.628, 0.631, 0.562, 0.527, and 0.482, respectively. When the cut-off score was equal to 5, the values of sensitivity, specificity, and the Youden’s index were 91.2, 76.6%, and 0.678, respectively (Table 4).

**Table 4:** Relevant indicators of PHQ-9 validity analysis

Cut-off value	Sensitivity	Specificity	Positive predictive value	Negative predictive value	Youden index
3	95.6%	55.5%	41.1%	97.5%	0.511
4	94.1%	68.4%	49.2%	97.3%	0.625
5	91.2%	76.6%	55.9%	96.4%	0.678
6	82.4%	80.4%	57.7%	93.3%	0.628
7	77.9%	85.2%	63.1%	92.2%	0.631
8	69.1%	87.1%	63.5%	89.7%	0.562
9	61.8%	90.9%	68.9%	88.0%	0.527
10	54.4%	93.8%	74.0%	86.3%	0.482

### Cut-off scores of PHQ-9 for depression with standard of HAMD-17

**Fig. 3:**
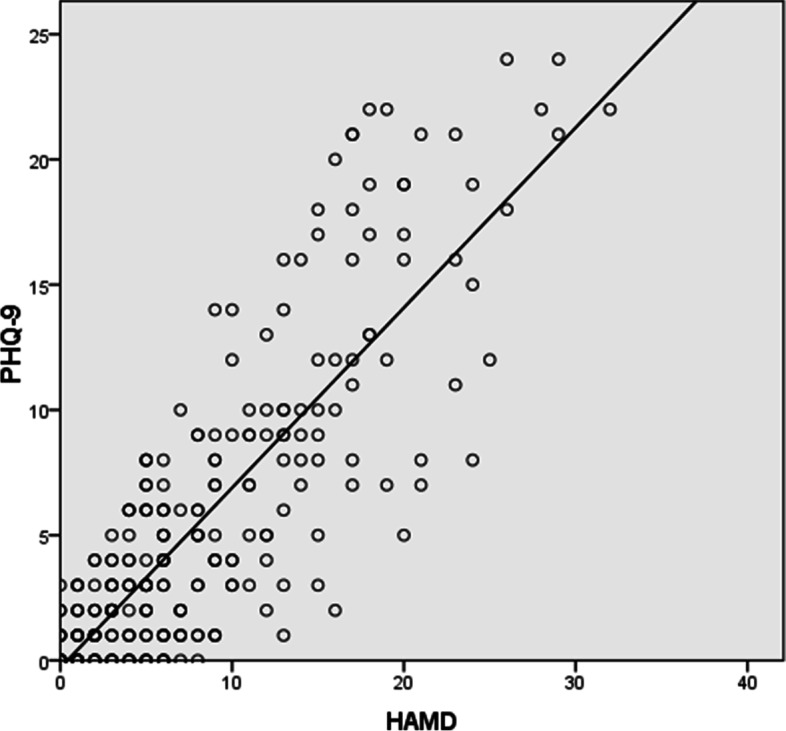
Consistency analysis between PHQ-9 and HAMD

The consistency analysis between PHQ-9 and HAMD showed a Kappa coefficient of 0.423. Using total score of HAMD as the independent variable, linear regression analysis of total score of HAMD and total score of PHQ-9 were performed (Fig. 3). Using the total score of HAMD as independent variable X and the total score of PHQ-9 as the dependent variable Y, the regression equation was Y = 0.719X - 0.299. The t-test was conducted on regression coefficient of 0.719 (*P* < 0.01), and regression relation was observed between the total HAMD score and total PHQ-9 score. The coefficient of determination R^2^ was equal to 0.701, and the regression model showed a good fit. Cut-off points of 7, 17, and 24 on HAMD scale represented mild, moderate, and severe symptom levels; the corresponding cut-off points on PHQ-9 scale were 5, 12, and 17, respectively.

### Consistency analysis

#### Consistency analysis of PHQ-9 and MINI

It was previously reported that in Chinese version of the PHQ-9, a threshold of 10 or more is an accurate, reliable, and valid measure for screening depressive symptoms. Thus, taking 10 as cut-off score of the PHQ-9, a consistency analysis of the results of PHQ-9 and the MINI was conducted, and the Kappa value was 0.529, *P* < 0.01. However, with taking 5 as the cut-off score of the PHQ-9, the consistency analysis with the MINI showed that the Kappa value was 0.558, *P* < 0.01.

#### Consistency analysis of PHQ-9 and HAMD assessment

Severe and extremely severe cases of depression, as rated by the PHQ-9, were unified as severe. We used the cut-off values of 5, 10, and 15 for mild, moderate, and severe depression, and the depression rating scores derived from PHQ-9 and from HAMD were evaluated for consistency. The consistency analysis between PHQ-9 and HAMD showed a Kappa coefficient of 0.423, *P* < 0.01. In this study, we used the cut-off scores 5, 12, 17, for mild, moderate, and severe depression derived from PHQ-9 as variables, Kappa = 0.465, P < 0.01.

### Analysis of depression-associated factors

Univariate analysis of depressive patients with neurological disorders who were hospitalized in department of neurology was carried out by using the Chi-square test, and the results are summarized in Table 5. The effects of gender, age, marital status, ethnicity, work, expenses of hospitalization, course of disease, and major diseases in the depression and non-depression groups were not statistically significant.

**Table 5:** Univariate analysis of depression with inpatients in the Department of Neurology

Factor	Total	Depresssion		Non-depressive		χ^2^	*P*
		num	Ratio(%)	num	Ratio(%)		
**Gender**	277	68		209		1.691	0.193
Male	181	40	58.8	141	67.5		
Female	96	28	41.2	68	32.5		
**Age (year)**	277	68		209		1.010	0.603
18 ~ 40	28	6	8.8	22	10.5		
41 ~ 65	124	34	50.0	90	43.1		
> 65	125	28	41.2	97	46.4		
**Marriage**	277	68		209		2.374	0.499
Married	252	61	89.7	191	91.4		
Divorce	2	0	0	2	1.0		
Widowed	4	2	2.9	2	1.0		
Unmarried	19	5	7.4	14	6.7		
**Ethnicity**	277	68		209		0.000	1.000
Han	264	65	95.6	199	95.2		
Other ethnicities	13	3	4.4	10	4.8		
**Work status**	277	68		209		7.177	0.066
Public servants	83	14	20.6	69	33.0		
Retirement	112	26	38.2	86	41.1		
Unemployed	29	11	16.2	18	8.6		
Others (students, farmers, etc.)	53	17	25.0	36	17.2		
**Medical expenses**	277	68		209		0.970	0.325
Reimbursement	271	65	95.6	206	98.6		
Own expense	6	3	4.4	3	1.4		
**Major disease**	277	68		209		7.636	0.177
Cerebral infarction	161	45	66.2	116	55.5		
Cerebral hemorrhage	15	3	4.4	12	5.7		
Transient ischemic attack	10	1	1.5	9	4.3		
Posterior circulation ischemia	23	3	4.4	20	9.6		
Peripheral neuropathy	14	1	1.5	13	6.2		
Other disease (epilepsy,cervical spondylosis, etc.)	54	15	22.1	39	18.7		
**Number of diseases**	277	68		209		1.869	0.388
1 ~ 3	49	12	17.6	37	17.7		
4 ~ 6	124	26	38.2	98	46.9		
> 7	104	30	44.1	74	35.4		
**Course of disease**	277	68		209		0.705	0.703
Less than 1 month	205	50	73.5	155	74.2		
1 month to 1 year	42	9	13.2	33	15.8		
More than 1 year	30	9	13.2	21	10.0		

Multivariate logistic regression analysis showed that the influence of unemployment on the occurrence of depression was statistically significant (*P* = 0.027, odds ratio (OR) = 3.080, 95%CI: 1.133 ~ 8.374). As shown in Table 6, unemployed patients were at a high risk of depression compared with employed patients.

**Table 6:** Multivariate analysis of depression in inpatients in the Department of Neurology

Variable	Standard	β	Waldχ^2^	*P*	OR	95%CI	
						Lower	Upper
**Gender**	Male	0.445	1.973	0.160	1.561	0.839	2.906
**Age (**year)	18 ~ 40		1.290	0.525			
	41 ~ 65	0.534	0.512	0.474	1.705	0.396	7.351
	> 65	0.208	0.065	0.799	1.231	0.250	6.059
**Marriage**	Married		1.299	0.729			
	Divorce	−19.180	0.000	0.999	0.000	0.000	–
	Widowed	1.201	1.132	0.287	3.322	0.364	30.313
	Unmarried	0.302	0.156	0.693	1.353	0.302	6.064
**Ethnicity**	Han	−0.053	0.005	0.941	0.948	0.231	3.887
**Work**	Public servants		6.240	0.101			
	Retirement	0.394	0.869	0.351	1.483	0.648	3.398
	Unemployed	1.125	4.860	0.027^*^	3.080	1.133	8.374
	Others (students, farmers, etc.)	0.832	3.464	0.063	2.299	0.957	5.524
**Fee**	Reimbursement	1.751	2.939	0.086	5.759	0.778	42.607
**Major disease**	Other (epilepsy,etc.)		6.931	0.226			
	Cerebral infarction	0.270	0.230	0.632	1.311	0.434	3.958
	Cerebral hemorrhage	−0.126	0.021	0.885	0.881	0.160	4.852
	Transient ischemic attack	−1.056	0.790	0.374	0.348	0.034	3.572
	Posterior circulation ischemia	−0.842	1.195	0.274	0.431	0.095	1.950
	Peripheral neuropathy	−2.072	2.886	0.089	0.126	0.012	1.375
**Number of diseases**	1 ~ 3		3.497	0.174			
	4 ~ 6	−0.572	1.465	0.226	0.564	0.223	1.425
	7	0.025	0.003	0.959	1.026	0.392	2.687
**Course of disease**	< 1 month		0.182	0.913			
	1 month to 1 year	0.212	0.150	0.699	1.237	0.422	3.625
	> 1 year	0.217	0.116	0.733	1.242	0.358	4.310

Note: * *P* < 0.05

## Discussion

Depression is a widespread mental disorder that can pose threat to thoughts, mood, and physical health [33]. Depression severity was classified into three levels, including mild, moderate, and severe. Individuals with depression not only often experience sadness, but also a lack of interest or enjoyment in activities, decreased energy, insomnia, weight changes, feelings of loss and worthlessness, and recurrent thoughts of death or suicide. The prevalence of depressive disorders was higher in neurology inpatients [34, 35]. Our study found that a Chinese version of the MINI was used to assess the status of inpatients with neurological disorders admitted to the Department of Neurology of Peking University Third Hospital, and the results showed that the prevalence of depression was 24.5%, which was similar to outpatients in different clinical specialties, but significantly higher than outpatients in healthy controls [31]. This indicates that further attention should be paid to depression in non-psychiatric departments (e.g., department of neurology) of general hospitals.

PHQ-9, a universal community screening tool for depression, was herein used, and it was revealed that it had a good reliability and validity when it was applied to depressed patients with neurological disorders who were hospitalized at the department of neurology. It is noteworthy that the DSM-5 also recommends use of PHQ-9 as a tool for evaluating the severity of depression.

Studies conducted in China as well as overseas have consistently shown that PHQ-9 has an I-factor structure, i.e., affective factor; in other words, all items in PHQ-9 measure the same concept [36, 37]. Other studies have reported that PHQ-9 has II-factor structure: cognitive-affective factor and somatic factor [38]. In the current research, the structure validity of the PHQ-9 was analyzed by principal component analysis, and the results extracted three main factors contributing to a cumulative explained variance of 63.114%. The analysis of the three main factors was mainly related to low mood, lack of motivation and somatic symptoms. When the PHQ-9 was compared with the MINI, it outperformed with a reasonable accuracy in identifying cases of depression. The value of AUC was 0.898, suggesting a promising diagnostic ability of the PHQ-9. In a systematic review of PHQ-9, Kroenke et al. showed that the sensitivity was 77 - 88% and the specificity was 88 - 94% with 10 points as the cut-off value [39]. Importantly, the values of sensitivity obtained in this study was not as high as those reported by Kroenke et al. [39].There lies several reasons. (1) It may be related to the different source of subjects. (2) there may have another reason that when PHQ-9 is used to screen depression with patients in neurology, its sensitivity may be suboptimal and still needs further evaluation by related professionals. (3) What’s more, it might be related to the use of the MINI as a gold standard. A recent meta-analysis showed that the sensitivity of the PHQ-9 was lower (0.77 versus 0.88) when using the MINI as the gold standard compared to semi-structured interviews [40]. In the present study, there was a strong correlation between the total scores of HAMD-17 and PHQ-9, which was consistent with previous findings [41, 42]. These findings support the validity and feasibility of the use of PHQ-9 for assessing depression severity. In the current study, we used PHQ-9 scale scores of 5, 12, and 17 as cut-off scores to designate mild, moderate, and severe symptoms of depression, respectively. This is slightly different from the cut-off scores used by the original developers of the scale. They recommended cut-off scores of 5, 10, 15, and 20 to designate mild, moderate, moderately severe, and severe depression, which is also more easily remembered by clinicians.

Consistent with previous studies, the results of the present study revealed that the PHQ-9 has a high reliability evidenced by the Cronbach’s α coefficient. The internal consistency of the PHQ-9 was assessed by using the Cronbach’s alpha coefficient, and it was found to be 0.839. The correlation coefficients between the nine entries of the scale were 0.160 ~ 0.578 (P < 0.01), and the correlation coefficients between each entry and the total score of the scale were 0.608 ~ 0.773, all of which had a significant correlation relationship (*P* < 0.01). This indicates that the PHQ-9 has an acceptable predictive performance. Our findings were similar to those observed in validation studies whose Cronbach alpha values were found to be 0.8 in a study of Mexico [43], 0.74 in Australia [44], and 0.78 in Thailand [45].

The present study analyzed factors influencing depression in patients with neurological disorders who were hospitalized in department of neurology. We compared the factors of gender, age, marital status, ethnicity, work experience, hospitalization expenses, course of disease, number of patients, and major disorders in the depression and non-depression groups. The results of univariate analysis did not indicate any statistical significance. Previous studies reported that age and gender are significantly correlated with the occurrence of depression [46, 47]. It was previously found that the scores of depressive symptoms in stroke patients who aged 25-54 to 55-64 years old were significantly higher than those in other age-based groups [48]. It should be noted that the results of the current study did not reveal any significant correlation between depression and age/sex. The influences of age and gender on the depression of patients with neurological disorders who admitted to the department of neurology need further discussion. The current study did not make a detailed classification and comparison of various domestic reimbursement methods. The multivariate logistic regression analysis showed that unemployed cases were at a higher risk of depression. Previous studies showed that depression is closely correlated to unemployment. Scholars [49] pointed out that nearly one fifth of long-term unemployed men were diagnosed with major depressive disorders. Unemployment may be a potential predictor of depression, weakening the work productivity, thereby increasing the risk of long-term unemployment [50–52].

### Limitations

The application of PHQ-9 scale on such patients showed a good reliability and validity. However, the current study contains a number of limitations. First, the samples were only patients with neurological disorders from one general hospital. Second, due to the short period of hospitalization, no retesting of reliability was undertaken. Last but not least, this study did not analyze the effects of various neurological diseases on depression. Thus, further studies need to be carried out to confirm our findings and eliminate the above-mentioned deficiencies.

## Conclusions

In summary, depressive disorders are more common among patients with neurological disorders. Since depression can bring many adverse prognoses to patients, even lead to suicide, early identification of depression needs the attention of non psychiatrists. Our study demonstrated good reliability and validity of the PHQ-9 by applying this questionnaire to screen depressed patients in a neurology department of general hospital. PHQ-9 is worth promoting and applying in the general hospital department of neurology.

## Data Availability

The datasets used and/or analysed during the current study are available from the corresponding author on reasonable request.

## Abbreviations

PHQ-9: Patient Health Questionnaire-9
MINI: Mini-International Neuropsychiatric Interview
HAMD: Hamilton Rating Scale for Depression
ROC: Receiver Operating Characteristic
CI: Confidence Interval
SDS: Self-rating Depression Scale
SCID-I: Structured Clinical Interview for DSM-IV Axis I Disorders
CIDI: Composite International Diagnostic Interview
CSDD: Cornell Scale for Depression in Dementia
DSM-5: Diagnostic and Statistical Manual of Mental Disorders, Fifth Edition
DSM-IV: Diagnostic and Statistical Manual of Mental Disorders, Fourth Edition
ICD-10: International Classifications of Diseases and Related Health Problems, Tenth Revision
ICC: Intraclass Correlation Coefficient
AUC: Area under the ROC curve
OR: Odds Ratio
